# Adalimumab Autoantibodies in Uveitis Patients: Do We Need Routine Drug Monitoring?

**DOI:** 10.3390/biomedicines12122782

**Published:** 2024-12-06

**Authors:** Lynn S. zur Bonsen, Vitus A. Knecht, Anne Rübsam, Dominika Pohlmann, Uwe Pleyer

**Affiliations:** 1 Department of Ophthalmology, Charité—Universitätsmedizin Berlin, Corporate Member of Freie Universität Berlin and Humboldt—Universität zu Berlin, Augustenburger Platz 1, 13353 Berlin, Germany; 2 Berlin Institute of Health at Charité, Universitätsmedizin Berlin, Charitéplatz 1, 10117 Berlin, Germany

**Keywords:** adalimumab, antidrug antibodies, anti-TNF, therapeutic drug monitoring, non-infectious uveitis

## Abstract

Background: Adalimumab, an anti-TNF-α biologic agent, has emerged as a principal treatment option for patients with non-infectious uveitis. The influence of adalimumab anti-drug antibodies (AAA) on the efficacy of adalimumab therapy is not yet fully understood. We aim to understand their clinical implications in the context of therapeutic drug monitoring and the factors contributing to the formation of these antibodies. Methods: We conducted a retrospective analysis of 114 patients with non-infectious uveitis who developed AAA while undergoing adalimumab therapy. Results: Among the 114 AAA-positive uveitis patients, a significant correlation was observed between AAA levels and reduced adalimumab serum levels (r = −0.58, *p* < 0.001). The mean time to AAA detection was 2.1 years (range 0.1–11.9 years), with 45.6% of cases identified through routine testing. If AAA levels were initially low, subsequent measurements for AAA were more likely to become negative during treatment (r = 0.63, *p* < 0.001). Higher AAA concentrations were associated with a shorter time to detection (r = −0.27, *p* = 0.01) and younger age (r = −0.21, *p* = 0.03). There was a trend, though no significant influence, of concomitant immunosuppression with prednisolone ≤ 7.5 mg or methotrexate on antibody formation (*p* = 0.18). No significant difference was observed in AAA levels between uveitis subtypes. Conclusions: Higher AAA concentrations are associated with lower adalimumab serum levels in uveitis patients. Routine clinical testing is essential for optimal therapeutic drug monitoring to prevent early loss of effectiveness.

## 1. Introduction

Biological disease-modifying anti-rheumatic drugs (DMARDs) have revolutionized the therapy of non-infectious uveitis (NIU), an autoimmune eye disease that can potentially result in irreversible vision loss in up to 20% of patients [1]. While immunosuppressants are the primary treatment for NIU, the significant side effects associated with steroids necessitate a transition to long-term DMARD therapy for patients with persistent inflammation. Given the limitations of conventional DMARDs, a switch to biological agents is the next step on the therapeutic ladder. Among these, adalimumab, the only FDA-approved human monoclonal antibody for uveitis, is the most commonly used [2,3]. However, the development of adalimumab anti-drug antibodies (AAA) as a consequence of immunogenicity can result in a reduction of the drug’s effectiveness.

Due to their protein nature, biological agents, including adalimumab, have the potential to elicit an adaptive immune response. The immunogenicity of these agents is contingent upon their size and structural characteristics. As a human antibody, adalimumab is, therefore, capable of exhibiting a certain degree of immune tolerance. However, the formation of autoantibodies against the idiotype in the binding region has been observed, which results in a loss of efficacy of adalimumab [4]. Initially, immunoglobulin M (IgM) antibodies are produced, which are subsequently replaced by immunoglobulin G (IgG) antibodies as a result of the stimulation of B cells. The affinity of these antibodies to the receptors may vary between patients, depending on the individual’s immunological response [5]. Adalimumab appears to be more immunogenic than other TNF inhibitors, such as golimumab or etanercept. The observed variation can be attributed to inherent differences in the pharmacological compound and its mechanism of action [6].

Monitoring AAA formation is vital for managing adalimumab therapy in NIU, yet it remains a debated topic. Routine therapeutic drug monitoring (TDM), which involves systematic screening of all patients, could facilitate the early detection of AAA, potentially enabling the timely adjustment of therapy prior to the manifestation of clinical symptoms. Conversely, selective testing may optimize the use of resources and focus efforts on patients with specific clinical indicators, such as increased disease activity or the development of macular edema.

This study hypothesized that routine monitoring (proactive) may be more effective than selective testing (reactive) in the long-term management of adalimumab treatment despite its higher initial resource demands. The potential benefit of routine testing lies in its ability to detect AAA formation earlier, potentially preventing clinical deterioration and improving overall treatment efficacy.

The risk of developing AAA is associated with both patient-related and drug-related factors. Given the diverse nature of uveitis, which encompasses a multitude of pathogenetic pathways, it is important to investigate whether antibody formation is more prevalent in patients with systemic immune diseases in comparison to those with purely ocular autoimmune uveitis. Moreover, there is a strong association between the female gender and an increased risk of developing autoimmune diseases. It remains unclear whether gender and age influence the formation of AAA. Furthermore, the administration of adalimumab also affects the formation of antibodies. A study of rheumatology patients demonstrated that a temporary interruption of therapy was associated with an increased risk of AAA, while concomitant methotrexate (MTX) application provided a protective effect [7]. From a clinical perspective, it is necessary to determine how adalimumab therapy should be adjusted based on the occurrence of AAA formation while acknowledging the potential for transient antibody formation [8].

To date, there have been only very few studies investigating the presence of AAA in patients with NIU. Therefore, the aim of the present study is to investigate the relationship between the formation of AAA and adalimumab drug levels in patients with NIU. The objective is to evaluate the effectiveness of routine diagnostics in comparison to selective testing for monitoring AAA, with the purpose of optimizing the long-term management of adalimumab therapy. Furthermore, the study aims to identify potential risk factors related to demographic data, different forms of uveitis, and therapeutic characteristics.

## 2. Materials and Methods

### 2.1. Study Population

In this single-center study, we retrospectively analyzed the medical records of patients with NIU who were treated with adalimumab and tested positive for AAA. The analysis covered the period from September 2021 to July 2023. The study was conducted in accordance with the Declaration of Helsinki after receiving approval from the local ethics committee (EA4/047/24).

Uveitis was classified according to the Standardization of Uveitis Nomenclature (SUN) criteria, including the localization of uveitis, the subtype of ocular inflammation, and any associated systemic diseases.

Adalimumab (HUMIRA^®^, AbbVie, Ludwigshafen, Germany) was administered subcutaneously at a dose of 40 mg every 2 weeks. In standard practice, an initial loading dose of 80 mg is given, followed by a 40 mg dose one week later. The therapy is generally well tolerated, although the most frequent adverse events include injection-site reactions and respiratory tract infections. Less common adverse events reported involve allergic reactions, severe infections, or tumors [2]. Adalimumab is contraindicated in patients with a known hypersensitivity to the drug or its components, active tuberculosis, or other severe infections. Furthermore, it must be avoided in those with moderate to severe heart failure.

Data collected included the initiation date of adalimumab therapy, the reason for the antibody test, any induction phases or interruptions in therapy, and whether additional antibody tests were performed. The use of immunosuppressive systemic therapy prior to and during adalimumab administration was documented.

### 2.2. Laboratory Testing

Serum levels of adalimumab and AAA were quantified through the use of an enzyme-linked immunosorbent assay (ELISA). The detection threshold for AAA was set at 0.6 AU/mL. Patients were routinely monitored at each visit during their adalimumab therapy, with the typical monitoring interval set at 3 to 6 months. Additionally, AAA testing was conducted for the following reasons: suspected recurrence of uveitis inflammation, recurrence of macular edema, persistent inflammation despite ongoing treatment, and persistent macular edema. For individuals tested more than once during the study period, only the first positive AAA result was included in the analysis. Subsequent AAA results were also assessed to determine whether they remained positive or became negative. Adalimumab therapy was continued even after AAA was detected, based on clinical judgment, taking into account the patient’s clinical response, as well as the serum levels of AAA and adalimumab. Alternatively, the therapy of the patients was switched.

### 2.3. Statistical Analysis

Prism GraphPad statistical software (version 10.1.2., GraphPad Software, Boston, MA, USA) was used to analyze patient data from the electronic medical record system.

Continuous variables were expressed as mean ± standard deviation (SD). Categorical variables were presented as frequencies and percentages. Comparisons between groups were conducted using the Student’s *t*-test or Mann–Whitney U test for continuous variables and the chi-square test or Fisher’s exact test for categorical variables. Correlation analyses with metric variables were evaluated using either Pearson or Spearman correlation coefficients, as appropriate. A *p*-value of < 0.05 was considered statistically significant.

## 3. Results

### 3.1. Patient Characteristics

The study cohort comprised 114 patients with an average age of 42.5 years (range: 5–81 years). The cohort included a higher proportion of females, accounting for 61.4% of the patients. Uveitis was classified according to SUN criteria, resulting in the following distribution: 42 patients presented with posterior uveitis, 32 with intermediate uveitis, 24 with anterior uveitis, and 16 with panuveitis. The majority of patients (82.4%) exhibited bilateral involvement. The most prevalent ocular subtypes, in addition to idiopathic uveitis (n = 38), were punctate inner choroidopathy (n = 7), multifocal choroiditis (n = 6), and serpiginous choroiditis (n = 6). Systemic diseases were present in 43% of the patients, with ankylosing spondylitis (n = 12), sarcoidosis (n = 9), and juvenile idiopathic arthritis (n = 9) being the most common. Further details of the distributions can be found in Table 1.

### 3.2. Therapeutic Drug Monitoring

#### 3.2.1. Effect on Adalimumab Serum Levels

**Figure 1:**
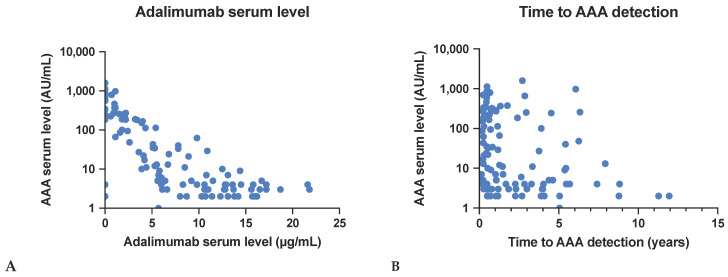
(**A**): Correlation between adalimumab serum levels and adalimumab anti-drug antibody (AAA) serum levels. This figure shows the moderate inverse relationship between adalimumab serum levels and AAA serum levels, indicating that higher AAA levels are associated with lower adalimumab levels. (**B**): Correlation between the years to the detection of AAA and AAA serum levels. The graph demonstrates that elevated AAA levels are frequently observed relatively early after adalimumab treatment initiation.

A moderate inverse relationship was observed between AAA serum levels and serum adalimumab levels. Higher AAA serum levels were significantly associated with lower adalimumab serum levels, with a correlation coefficient of r = −0.58 (*p* < 0.001). The mean AAA level was 158.1 AU/mL (±283.1) (range: 1.0–1584.0 AU/mL). The mean adalimumab serum level was 6.9 µg/mL (±5.8) (range: 0.0–21.8 µg/mL). See Figure 1A for a visual representation of these findings.

#### 3.2.2. Time to AAA Detection

A weak negative correlation was found between AAA serum levels and the time taken to detect AAA. Higher levels of AAA were associated with a shorter time to detect the antibodies, with a correlation coefficient of r = −0.27 (*p* = 0.01). The mean time to AAA detection was 2.1 ± 2.5 years. The correlation is shown in Figure 1B. 81 patients (71.1%) had previously undergone AAA testing and had yielded negative results. In contrast, 33 patients (28.9%) had not undergone any prior testing.

#### 3.2.3. Reason for AAA Testing

**Figure 2:**
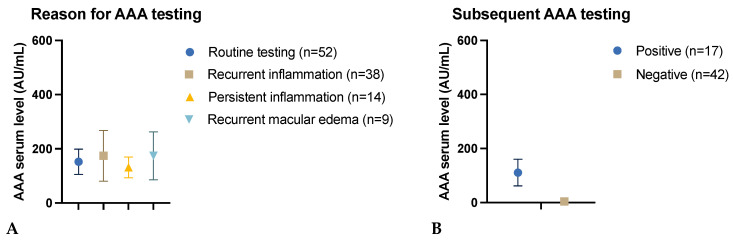
(**A**): AAA serum levels in relation to the reason for AAA testing. One case of persistent macular edema exhibited an AAA level of 43 AU/mL. (**B**): AAA serum levels in relation to the result of subsequent AAA testing. 55 patients are not presented as they have not been retested. AAA serum levels are presented as the mean ± standard error of the mean.

No statistically significant difference was found between the groups with regard to the rationale for AAA testing (*p* = 0.09). The mean AAA level in the group undergoing routine sampling (n = 52) was 152.2 AU/mL (±336.6), while in patients with recurrent inflammatory activity (n = 38), the mean AAA level was 175.3 AU/mL (±251.7). The mean AAA level for the group tested due to persistent inflammatory activity (n = 14) was 131.4 AU/mL (±143.8), while the group with recurrent macular edema (n = 9) exhibited a mean AAA level of 174.0 AU/mL (±280.8). One case of persistent macular edema exhibited an AAA level of 43 AU/mL (±0.0) (Figure 2A).

#### 3.2.4. Impact of the Initial Test Result on Subsequent AAA Testing

A positive correlation was observed between AAA serum levels and sustained positive antibody status after a period exceeding three months. Patients with a persistent positive antibody status (n = 17) exhibited a significantly higher mean AAA level of 110.8 AU/mL (±203.0) compared to those with a negative status over time (n = 42), who demonstrated a mean AAA level of 4.5 AU/mL (±2.9) (*p* < 0.001). A total of 55 patients were not retested (Figure 2B).

### 3.3. Factors Affecting AAA Formation

#### 3.3.1. Effect of Age

**Figure 3:**
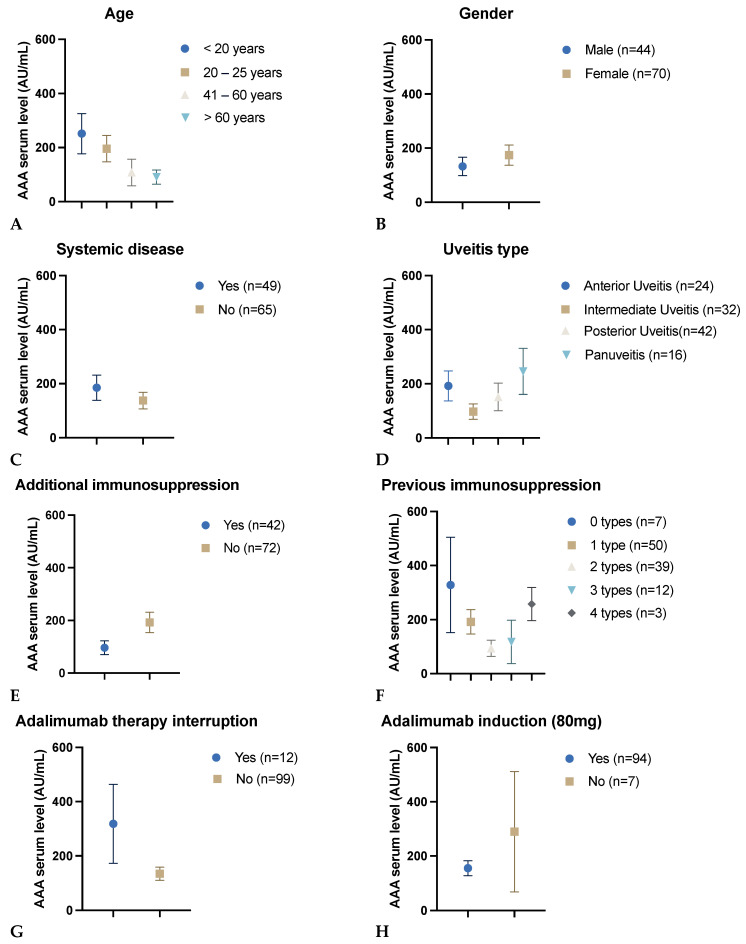
Serum levels of adalimumab anti-drug antibodies (AAA) in relation to age (**A**), gender (**B**), systemic diseases (**C**), uveitis types (**D**), additional immunosuppression (**E**), previous immunosuppression (**F**), adalimumab therapy interruption (**G**), and adalimumab induction dose (**H**). For graphs (**F**,**G**), no data was available for three patients each. For graph (**H**), there was incomplete information for 13 patients. AAA serum levels are presented as the mean ± standard error of the mean.

The analysis revealed a weak negative correlation between AAA serum levels and patient age. The cohort had a mean age of 42.5 years (±17.1) and a mean AAA level of 158.1 AU/mL (±283.2). The Spearman correlation coefficient was r = −0.21 (*p* = 0.03), indicating that younger patients exhibited higher AAA levels (Figure 3A).

#### 3.3.2. Effect of Gender

There was no significant difference in AAA serum levels between male (n = 44) and female (n = 70) patients (*p* = 0.38). The mean AAA level for male patients was 132.6 AU/mL (±225.6), while for female patients, it was 174.1 AU/mL (±314.5) (Figure 3B).

#### 3.3.3. Effect of Concomitant Systemic Disease

There were no statistically significant differences in AAA serum levels between patients with (n = 49) and without (n = 65) concomitant systemic autoimmune diseases (*p* = 0.33). The mean AAA level for patients with systemic disease was 185.4 AU/mL (±325.0), whereas for patients without systemic disease, it was 137.6 AU/mL (±247.6) (Figure 3C).

#### 3.3.4. Effect of Uveitis Type

No statistically significant differences in AAA serum levels were observed between various subtypes of uveitis (*p* = 0.37), indicating that the specific type of uveitis did not significantly impact AAA formation. The mean AAA level was observed to be lower in patients with intermediate uveitis (97.3 AU/mL ± 161.0), followed by 151.5 AU/mL (±333.1) in patients with posterior uveitis. The mean AAA level for patients diagnosed with anterior uveitis was 192.5 AU/mL (±271.8). The mean AAA level in patients with panuveitis was 245.7 AU/mL (±340.3), representing the highest mean value (Figure 3D).

#### 3.3.5. Effect of Additional Immunosuppression

There were no significant differences in AAA serum levels between patients with and without additional immunosuppression (*p* = 0.18), including prednisolone ≤ 7.5 mg or MTX. Patients who did not receive any additional immunosuppression (n = 72) had a mean AAA level of 194.3 AU/mL (±328.1). The mean AAA level for patients receiving prednisolone (n = 24) was 95.6 AU/mL (±155.4). For those treated with MTX (n = 17), the mean AAA level was 102.4 AU/mL (±193.3). The AAA levels appeared to be comparable between patients undergoing concomitant prednisolone or MTX treatment (*p* = 0.91). (Figure 3E).

#### 3.3.6. Effect of Previous Immunosuppression

The analysis revealed no statistically significant correlation between the number of prior drug classes utilized for immunomodulatory therapy and AAA serum levels (*p* = 0.14). The mean AAA level for patients who had not previously undergone immunosuppression was 328.3 AU/mL (±467.4). In comparison, patients who had used one class of previous immunosuppression exhibited a mean AAA level of 191.7 AU/mL (±318.6). Those with two classes of previous immunosuppression had a lower mean AAA level of 93.9 AU/mL (±189.5). Patients who had received three classes of immunosuppression had a mean AAA level of 117.6 AU/mL (±278.5), and those with four classes of previous immunosuppression had a mean AAA level of 257.7 AU/mL (±106.5) (Figure 3F).

#### 3.3.7. Effect of Adalimumab Interruption

Of the study participants, 12 patients paused their adalimumab therapy, while 99 patients were continuously treated. The reasons for treatment interruption included infection, missing prescription, discontinuation in the absence of inflammation, vaccination, and pregnancy. Those who discontinued their therapy exhibited a higher mean AAA serum level of 318.3 AU/mL (±504.1) compared to patients who did not pause, with a lower mean AAA level of 135.0 AU/mL (±241.3). This indicates a weak positive correlation between a break in adalimumab therapy and increased levels of AAA. However, the correlation between the variables was not statistically significant (correlation coefficient r = 0.14, *p* = 0.16) (Figure 3G).

#### 3.3.8. Effect of Adalimumab 80 Mg Induction

There were no statistically significant differences in AAA serum levels between patients with (n = 94) and without (n = 7) adalimumab 80 mg induction dose (*p* = 0.25). The mean AAA level for patients with 80 mg induction dose was 155.2 AU/mL (±264.5), whereas for patients without 80 mg induction dose it was 289.6 AU/mL (±586.6) (Figure 3H).

## 4. Discussion

Adalimumab, a human monoclonal antibody, represents a pivotal component in the management of NIU. This is especially notable given that it is the sole FDA-approved biological agent for the long-term treatment of NIU. The emergence of AAA can compromise the drug’s efficacy, underscoring the need for effective monitoring strategies to ensure the maintenance of consistent therapeutic drug levels. The present study offers insights into the utility of testing strategies and factors associated with the formation of AAA.

The VISUAL I study, which assessed adalimumab therapy over a maximum of 48 weeks, found an incidence of AAA formation at 2.7% [2]. In contrast, a meta-analysis of data from patients treated with adalimumab for NIU revealed a higher prevalence of 9% for AAA [9]. Real-world analyses have demonstrated significantly higher frequencies of up to 35% compared to the clinical trials [10]. These findings highlight the importance of considering real-world data to understand the actual frequency of AAA formation and its impact on treatment efficacy [7].

### 4.1. Therapeutic Drug Monitoring

The high prevalence of AAA raises the question of whether routine testing for these antibodies would be beneficial. The 2022 recommendations from the European Alliance of Associations for Rheumatology (EULAR) only advocate for the use of reactive therapeutic drug monitoring [11]. However, there is a range of available biological agents for the treatment of other rheumatic diseases. In our study, we assessed the reasons for testing and found that inflammatory activity was the primary driver for testing in our cohort. Nevertheless, 45.6% of the tests were performed as part of routine procedures. No statistically significant differences in AAA levels were observed between the groups, indicating that AAA can be identified prior to any discernible loss of efficacy. This emphasizes the significance of drug monitoring throughout the course of therapy [12]. A prospective study on patients with immune-mediated inflammatory diseases demonstrated that therapeutic drug monitoring of infliximab therapy resulted in superior inflammation control after 52 weeks [13].

While the financial burden of routine AAA testing is considerable, it is necessary to consider the costs associated with antibody therapy in the absence of a systemic effect. Various studies have investigated the cost-effectiveness of drug monitoring. An observational study found a monthly cost saving of EUR 324 per patient. In addition, the interval could be significantly extended and the average dose reduced by approximately 20% by monitoring the drug level at the same time [14]. In addition to cost savings, other studies have also identified a notable enhancement in quality-adjusted life years (QALY) through the monitoring of adalimumab [15,16,17].

Our findings indicate that routine AAA testing could be beneficial in identifying AAA formation early, potentially averting clinical deterioration before it becomes apparent. This is consistent with the findings of previous studies [18], which have highlighted the variability in antibody responses and the need for individualized monitoring strategies. Routine testing might be more effective in preemptively detecting AAA and adjusting treatment strategies accordingly. Conversely, selective testing—triggered by clinical signs such as increased disease activity or macular edema—may result in delayed AAA detection, increasing the risk of disease progression and compromising outcomes. The challenge is to achieve an equilibrium between the advantages of early detection and the potential benefits of selective testing, which reflects a broader issue in clinical practice.

#### 4.1.1. Adalimumab Serum Level and Transient Antibodies

The results of our investigation indicate a correlation between AAA serum levels and reduced adalimumab drug levels. This finding is supported by a study that demonstrated that NIU patients without AAA had a mean adalimumab serum level of 13.6 (±5.2 μg/mL), while those with AAA formation had a mean adalimumab level of 2.8 (±2.6 μg/mL) [19]. Our results suggest that AAA levels are a key factor in treatment outcomes. In cases where AAA levels are low and declining, adalimumab therapy can still be continued successfully. Additionally, Cordero-Coma et al. described the formation of transient antibodies [8]. The presence of transient antibodies can be attributed to the process of tolerance development and B-cell anergy [5]. In regard to the timing of AAA testing, this is also a significant consideration. It is recommended that repeated testing be conducted, particularly in patients exhibiting low AAA levels. In this context, the weekly administration of adalimumab may prove beneficial in avoiding treatment resistance and preventing the necessity for a change in therapy [19].

#### 4.1.2. Timing of the Antibody Test

Another important consideration is the timing of the antibody test. The mean time to detection of AAA was 2.1 years. The interval between the commencement of adalimumab therapy and the onset of AAA exhibited considerable variability, with a range of 35 to 4359 days. These significant discrepancies in the time period have been corroborated by other studies [10,19]. A study of patients with ankylosing spondylitis demonstrated that early formation of antibodies and the reduction of drug levels four weeks after the initiation of therapy can predict a lack of response to therapy [20]. In our study, we found that patients under the age of 50 were particularly likely to have high levels of AAA in their serum. Furthermore, we observed that especially high AAA levels were associated with a shorter time to detect the antibodies. Consequently, patients at risk of high antibody levels can be identified by early antibody testing.

### 4.2. Factors Affecting AAA Formation

#### 4.2.1. Uveitis in Systemic Autoimmune Diseases

In contrast to our study, a meta-analysis identified systemic autoimmune diseases as a risk factor for AAA formation in NIU patients [9]. Moreover, antinuclear antibodies (ANA) have been identified as a risk factor in juvenile idiopathic arthritis (JIA) patients [21]. In our cohort, all JIA patients who tested positive for AAA also exhibited ANA. In a prospective study examining the development of infliximab anti-drug antibodies in individuals with other immune-mediated inflammatory diseases, several factors were identified as potential risk markers. These included lifetime smoking, a history of rheumatoid arthritis (RA), and a gap in treatment of over 11 weeks. Conversely, spondyloarthritis and higher serum drug levels appeared to have a protective effect [22].

#### 4.2.2. Additional Immunosuppression

Non-compliance with other immunosuppressants has been found to be associated with a higher risk for the occurrence of AAA [9,22]. The results of our study revealed no statistically significant differences between the two groups. However, there was a trend towards higher AAA levels in patients not receiving additional MTX or prednisolone ≤ 7.5 mg. Another working group was unable to demonstrate any correlation with concomitant use of other immunosuppressants [8]. Moreover, it is conceivable that previous immunosuppression may exert a protective effect against the formation of AAA. In our cohort, there was a trend towards lower AAA levels in patients with previous immunosuppression, although this did not reach statistical significance.

#### 4.2.3. Adalimumab Interruption

Patients who temporarily discontinued adalimumab therapy demonstrated higher AAA levels compared to those who remained on continuous treatment. This finding corroborates the hypothesis proposed by Bromeo et al., who identified therapy interruption as a risk factor for AAA formation [23]. The reasons for the pause of therapy included the following: infection, missed prescriptions, discontinuation in the absence of inflammation, vaccination, or pregnancy. This renders the decision to discontinue therapy for inactive uveitis patients particularly challenging, especially when there is an increased likelihood of AAA formation during a new flare and subsequent restart. Given the correlation between therapy interruptions and increased AAA levels, it would be prudent to conduct testing shortly after any treatment pause.

### 4.3. Limitations

The findings of the study must be considered in light of its inherent limitations. The first limitation of the study is that the trough levels of adalimumab were not measured. Consequently, the correlations of AAA levels with the various parameters were used. A further limitation of this study is its retrospective nature, which limits the ability to control for various confounding factors and introduces biases. For instance, there was a paucity of data regarding the incidence of vaccinations and infections in the medical records of the patients. In the case of routine testing, some of the measurements were only carried out at intervals of more than six months, given that patients were only able to adhere to longer intervals between examinations due to distance and time constraints. Moreover, the small sample size in certain subgroups may have reduced the statistical power of the results and limited their generalizability to a broader patient population.

## 5. Conclusions

In conclusion, our findings emphasize the importance of routine monitoring for AAA in NIU patients undergoing adalimumab therapy, particularly given the loss of effectiveness and variability in AAA formation. The prevention, early detection, and proactive management of AAA can significantly impact treatment efficacy and patient outcomes. This highlights the need for individualized and timely drug monitoring strategies.

## Figures and Tables

**Table 1:** Patient characteristics.

Characteristic	Mean ± SD ^1^
Age	42.5 years (±17.1)
Adalimumab anti-drug antibodies (AAA) serum level	158.1 AU/mL (±283.1)
Adalimumab serum level	6.9 µg/mL (±5.8)
Time to detection of AAA	2.1 years (±2.5)
Uveitis subtype	Number of patients
Posterior uveitis	42
Intermediate uveitis	32
Anterior uveitis	24
Panuveitis	16
Total	114
Specific ocular diseases	Number of patients
Punctate inner choroidopathy	7
Multifocal choroiditis	6
Serpiginous choroiditis	6
Birdshot retinochoroiditis	4
Sympathetic ophthalmia	3
Frosted branch angiitis	1
Acute zonal occult outer retinopathy	1
Acute posterior multifocal placoid pigment epitheliopathy	1
Idiopathic retinitis vasculitis aneurysms and neuroretinitis	1
Idiopathic	38
Systemic autoimmune diseases	Number of patients
Ankylosing spondylitis	12
Sarcoidosis	9
Juvenile idiopathic arthritis (ANA-positive)	9 (100%)
Vogt–Koyanagi–Harada disease	7
Behçet’s disease	6
Rheumatoid arthritis	5
Psoriasis	3
Tubulointerstitial nephritis and uveitis syndrome	1
Systemic lupus erythematosus	1

^1^ SD = standard deviation.

## Data Availability

The data that support the findings of this study are available from the corresponding authors, L.S.z.B. and V.A.K, upon reasonable request.
